# Cannibalism in *Tyrannosaurus rex*

**DOI:** 10.1371/journal.pone.0013419

**Published:** 2010-10-15

**Authors:** Nicholas R. Longrich, John R. Horner, Gregory M. Erickson, Philip J. Currie

**Affiliations:** 1 Department of Geology and Geophysics, Yale University, New Haven, Connecticut, United States of America; 2 Museum of the Rockies, Montana State University, Bozeman, Montana, United States of America; 3 Department of Biological Science, Florida State University, Tallahassee, Florida, United States of America; 4 Department of Biological Sciences, University of Alberta, Edmonton, Alberta, Canada; Raymond M. Alf Museum of Paleontology, United States of America

## Abstract

**Background:**

*Tyrannosaurus rex* was one of the largest terrestrial carnivores of all time, and consequently its ecology and diet have been the focus of much discussion. However, there is little direct evidence of diet or feeding habits in this species.

**Methodology/Principal Findings:**

Examination of museum collections has revealed four specimens of *Tyrannosaurus rex* that bear tooth marks made by large, carnivorous dinosaurs. Because *Tyrannosaurus* is the only large carnivore known from the Late Maastrichtian of western North America, we infer that *Tyrannosaurus* made these tooth marks.

**Conclusions/Significance:**

The marks are interpreted as feeding traces and these fossils therefore record instances of cannibalism. Given that this behavior has a low preservation potential, cannibalism seems to have been a surprisingly common behavior in *Tyrannosaurus*, and this behavior may have been relatively common in carnivorous dinosaurs.

## Introduction

The tyrannosaurids are a highly specialized group of carnivorous dinosaurs characterized by massive skulls, elongate hindlimbs, and highly reduced, didactyl forelimbs [1], [2], [3], [4], [5]. During the Late Cretaceous, they were the dominant large carnivores in North America and Asia [4], [5], with *Tyrannosaurus rex* being the last and the largest known member of the Tyrannosauridae [4], [5]. Indeed, it is one of the largest known terrestrial carnivores; weighing up to 10,000 kg [6] *Tyrannosaurus* was as large as the largest living land animal, the African elephant (*Loxodonta africana*), and it was comparable in size to the smallest baleen whale, the minke (*Balaenoptera acutorostrata*) [7]. By comparison, the largest living terrestrial hypercarnivore, the Siberian tiger (*Panthera tigris altaica*) weighs just 300 kg [7]. *Tyrannosaurus* is therefore radically different from any animal living today, or any creature that has existed in the past 66 million years. Unsurprisingly, the ecology of this remarkable animal has been the subject of considerable discussion, with particular emphasis placed on the issue of whether the animal was a predator, a scavenger, or both [4], [5], [8], [9], [10], [11], [12], [13]. Yet more than a century after the discovery of *Tyrannosaurus*, direct evidence of the animal's feeding habits is limited.

During recent museum studies of Maastrichtian dinosaurs, one of us (NRL) encountered a large theropod pedal phalanx (UCMP 137538) bearing tooth marks made by a large carnivorous dinosaur. Because *Tyrannosaurus* is the only large carnivore known from the late Maastrichtian of North America [14], the tooth marks and the phalanx can both be attributed to *Tyrannosaurus*. Subsequently, more dinosaur specimens have been found to bear *Tyrannosaurus* tooth marks, of which three are from *Tyrannosaurus* (Table 1). We show that these specimens provide direct evidence of cannibalism in *Tyrannosaurus*.

**Table 1:** Specimens showing tooth marks that are attributable to *Tyrannosaurus rex*, including previously described specimens (15) and specimens previously unidentified or unpublished (asterisk).

Taxon	Accession/Locality Number	Toothmarked element	Provenance
*Tyrannosaurus rex*	UCMP 137538*	pedal phalanx	Hell Creek Fm., Montana, late Maastrichtian
*Tyrannosaurus rex*	MOR 1126*	skeleton	Hell Creek Fm., Montana
*Tyrannosaurus rex*	MOR 920*	skeleton	Hell Creek Fm., Montana, late Maastrichtian
*Tyrannosaurus rex*	MOR 1602*	metatarsal III	Hell Creek Fm., Montana, late Maastrichtian
*Triceratops* sp.	YPM 53263*	squamosal	Lance Fm. Wyoming, late Maastrichtian
Ceratopsidae indet.	TMP 1998.102.0005*	frill fragment	Scollard Fm., Alberta, late Maastrichtian
Ceratopsidae indet.	MOR 799	pelvis	Hell Creek Fm., Montana, late Maastrichtian
Ceratopsidae indet.	NMC 53370*	ischium	Frenchman Fm., Saskatchewan, late Maastrichtian
Ceratopsidae indet.	UCMP 130385*	left dentary	Hell Creek Formation, Montana, late Maastrichtian
Ceratopsidae indet.	UCMP V86061*	limb bone fragment	Hell Creek Formation, Montana, late Maastrichtian
*Edmontosaurus annectens*	AMNH 5041*	dentary	Hell Creek Fm., Montana, late Maastrichtian
Hadrosauridae	UCMP 140601	pedal phalanx	Hell Creek Fm., Montana, late Maastrichtian
Hadrosauridae	UCMP uncatalogued*	metatarsal	Hell Creek Fm., Montana, late Maastrichtian
Hadrosauridae	CM 105*	pubis	Lance Fm., Wyoming, late Maastrichtian
Hadrosauridae	UCMP V86026*	caudal vertebra	Hell Creek Fm., Montana, late Maastrichtian
*Thescelosaurus neglectus*	MOR 1161*	Femur	Hell Creek Fm., Montana, late Maastrichtian
Ornithischia indet.	TMP 1994.125.0102*	Rib	Scollard Fm., Alberta, late Maastrichtian

## Materials and Methods

Fossils were examined at the American Museum of Natural History (AMNH), the Carnegie Museum of Natural History (CM); the Museum of the Rockies (MOR), the Canadian Museum of Nature (NMC), the Royal Saskatchewan Museum (RSM), the Royal Tyrrell Museum of Palaeontology, (TMP), the University of California Museum of Paleontology (UCMP), and the Yale Peabody Museum (YPM). These include the *T*. *rex* specimens described here (UCMP 137538, MOR 902; MOR 1126; 1602) as well as all *T*. *rex* specimens in the CM, RSM, TMP, UCMP, and YPM collections, although in some cases, not all elements of a skeleton were accessible for study.

## Results

**Figure 1:**
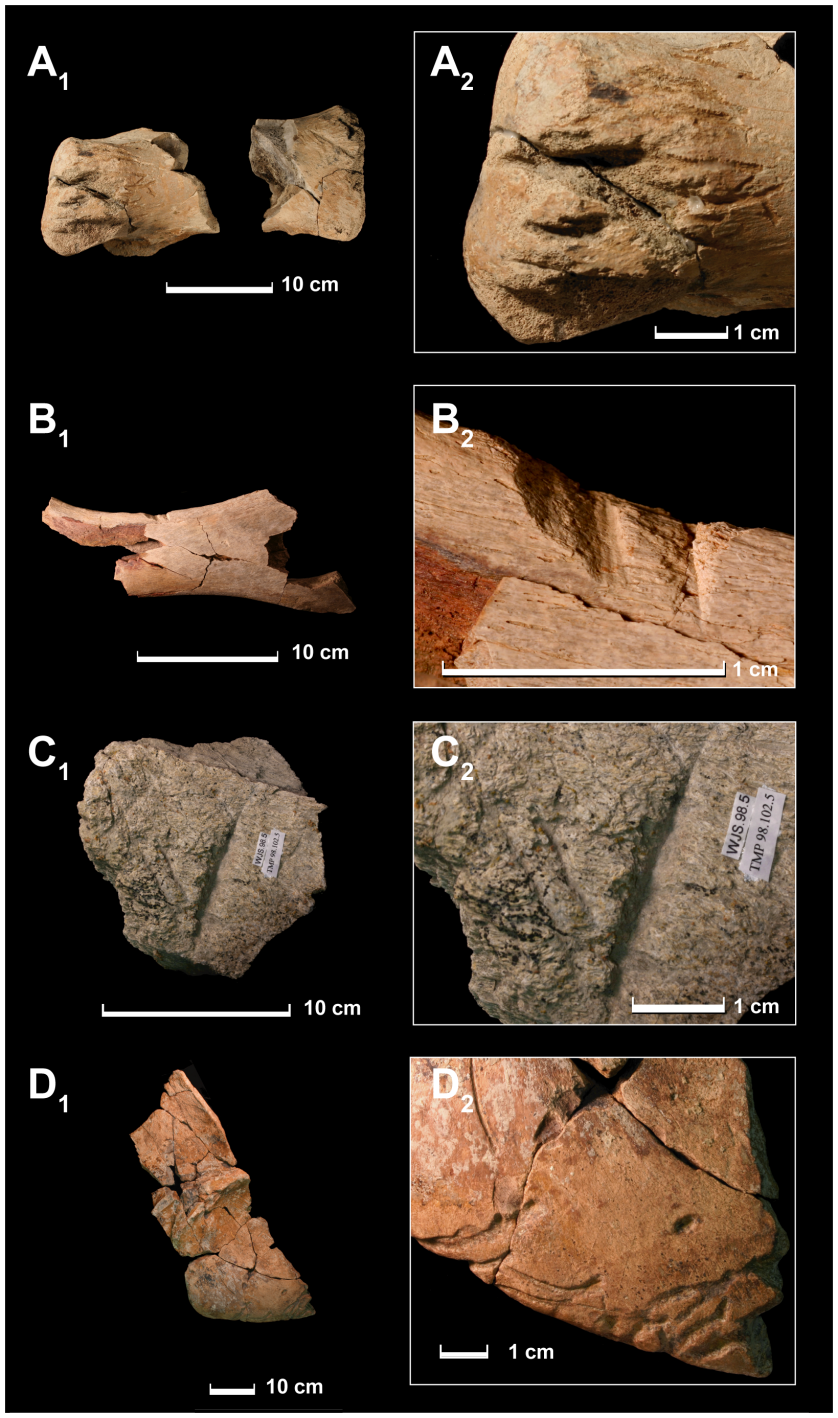
Tooth marks made by *Tyrannosaurus rex*. A, hadrosaurid metatarsal (UCMP uncatalogued) and closeup of tooth marks on distal articular surface. B, fragment of hadrosaurid pubis (CM 105) showing tooth marks on prepubic process. C, ceratopsid? frill element (TMP 1998.102.2) showing tooth mark. D, *Triceratops* right squamosal (YPM 53263) showing tooth marks on edge.

Including previously described specimens, a total of 17 specimens are identified as bearing tooth marks made by *Tyrannosaurus* (Figure 1; Table 1). These traces consist of deep U- and V-shaped gouges and shallower scores. None of the traces described here resemble the puncture marks found on a pelvis of *Triceratops*, but they closely resemble the furrowed ‘puncture and pull’ traces that have previously been attributed to *T*. *rex* [10], [15].

**Figure 2:**
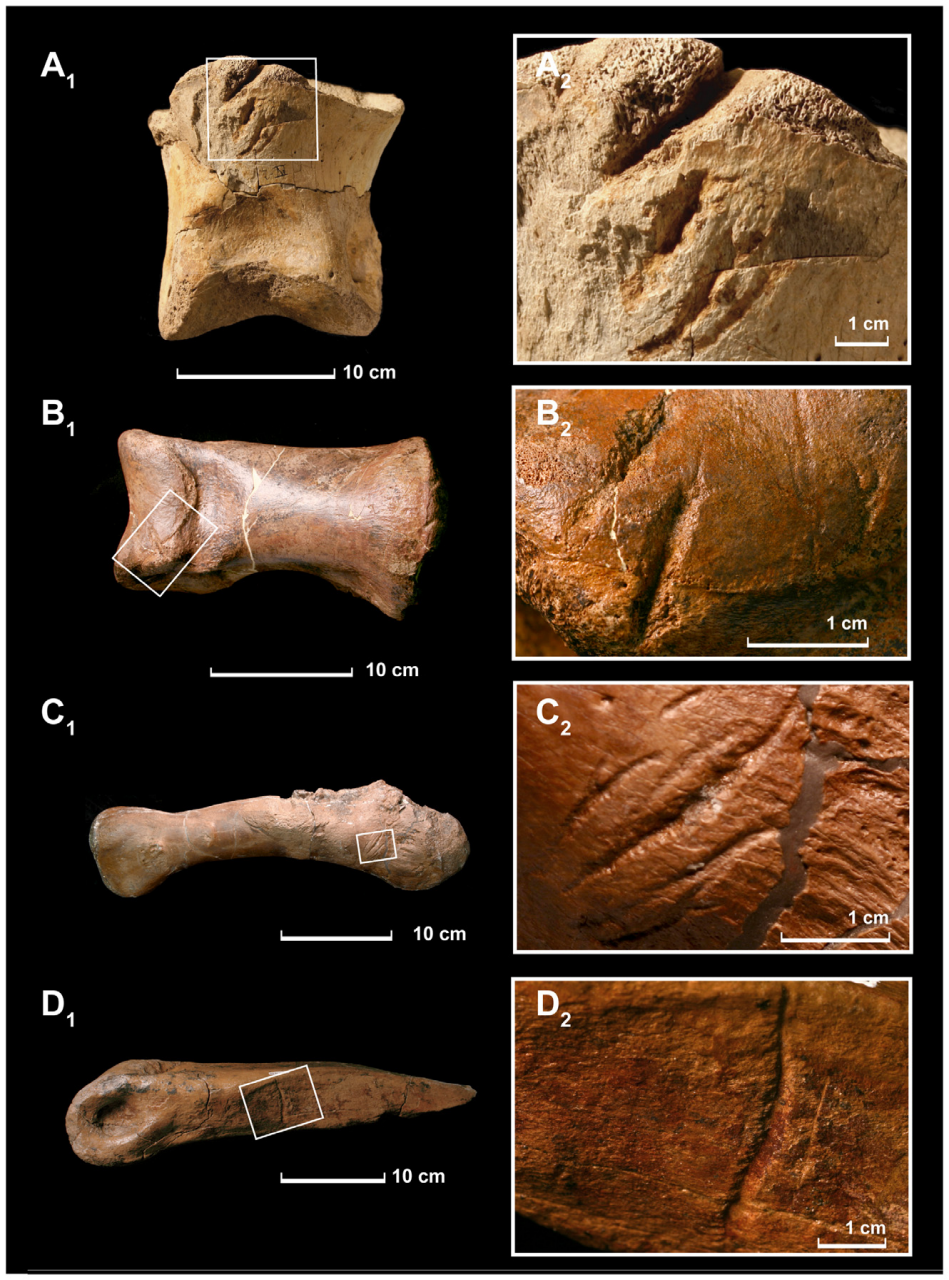
*Tyrannosaurus rex* bones bearing tooth marks made by *Tyrannosaurus rex*. A1, A2: UCMP 137538, pedal phalanx in dorsal view. B1, B2: Pedal phalanx, MOR 1126, dorsal view. C1, C2, Humerus of MOR 902 in caudal view. D1, D2 metatarsal III of *T*. *rex* MOR 1602, medial view.

Of these sixteen specimens, four represent *Tyrannosaurus* [Fig. 2]. The first is UCMP 137538, a large (13 cm long) pedal phalanx found in isolation (Fig. 2A). It is identified as a theropod by the gynglymous articular surfaces and deep collateral ligament pits, and is referred to *Tyrannosaurus* on the basis of its large size, robust construction, and provenance. Comparisons with FMNH PR 2081 [3] show that the bone is a left pedal phalanx IV-2 from a large, adult animal. The proximal end bears four gouges dorsally, and one ventrally, oriented at an oblique angle relative to the axis of the bone. The largest tooth mark is 25 mm long and 7 mm wide.

A second set of *Tyrannosaurus* bite marks was found on MOR 1126, a partial skeleton of *Tyrannosaurus*. On the left foot, pedal phalanx II-2 (Fig. 2B) bears gouges on the dorsal, lateral, medial, proximal, and distal surfaces. In one tooth mark, long striae left by denticles run through the gouge. The largest tooth mark is on the distal articular surface; it is approximately 70 mm long and 3.5 mm wide. Again, the bone comes from a large adult.

The third example comes from MOR 920, an associated skeleton of an adult *Tyrannosaurus*. The left humerus (Fig. 2C) bears a series of scores on its posterior surface. They are up to 36 mm long and 3 mm wide.

A fourth *Tyrannosaurus* specimen bearing conspecific bite marks is MOR 1602. The specimen consists of an isolated right metatarsal III missing the proximal half of the shaft (Fig. 2D). It is identified as *Tyrannosaurus* by its large size, the triangular shaft, and the splint-like proximal end. It bears two scores on medial surface; the larger is 44 mm by 4 mm. The distal end of the bone is approximately 11 cm across; again the bone is from a large adult.

## Discussion

The scores and gouges described here closely match the tooth marks of *Tyrannosaurus* [10], [15], and other theropods [16] in having a U to V-shaped section that tapers at either end. Given that the tooth marks are relatively deep and narrow, they were probably made by the laterally compressed teeth of the dentary and maxilla, rather than the incisiform premaxillary teeth; the tightly packed premaxillary teeth would also be expected to have left a series of closely spaced scores. An exception is UCMP 137538, where two closely spaced, subparallel gouges are found; these could conceivably have been made by premaxillary teeth.

It is extremely unlikely that any animal other than a theropod could have produced these traces. Crocodylians would not have produced the tooth marks described here. Whereas the serrated, laterally compressed teeth of theropods carve down into bone (as seen here) the subconical, unserrated teeth of crocodylians produce shallower score marks [17] or deep pits where the tooth punctures the bone [17]; such puncture marks are absent in the bones described here. Some lizards do have ziphodont teeth that can produce tooth marks resembling theropod tooth marks, notably *Varanus komodensis* [18]. The Hell Creek and Lance do contain a large lizard with ziphodont dentition, *Palaeosaniwa* [19]. However, even tooth marks made by the large *V*. *komodoensis* rarely exceed 1 mm in width [18]. *Palaeosaniwa* was considerably smaller than *V*. *komodoensis*, probably between 1 and 2 meters long, and therefore too small to have produced the traces described here. Mammals are known to gnaw on dinosaur bone [20], but mammalian gnaw traces are far smaller, and consist of closely spaced, paired tooth marks.

Insects can modify bone, but traces left by dermestid beetles are small and characterized by minute scratches left by the mandibles; termites produce meandering tunnels [21]. The large trace fossil *Cubiculum* [22], which is made by burrowing mayfly nymphs [23], (rather than carrion beetles), is common in the Hell Creek and Lance formations (NRL, pers. obs.) but consists of broad channels with a U-shaped section, which do not resemble the traces described here. Neither can these marks be accounted for by nonbiological mechanisms: trample marks are often seen on bones, but these consist of numerous small and closely spaced grooves (NRL, pers. obs.). These traces do not represent tool marks made during excavation, because tools could not penetrate deeply into the fossil without shattering the brittle bone.

It is highly unlikely that a non-tyrannosaurid theropod could have made the bite marks described here. Dromaeosaurids and troodontids are known from the late Maastrichtian of North America, but these are relatively small animals [24]. Given that tooth scores made by the much larger dromaeosaurid *Deinonychus* are just 1 mm wide [25] the small deinonychosaurs in the fauna could not have made the traces described here. Furthermore, bones bitten by dromaeosaurids are extremely rare, [25] and dromaeosaurid teeth exhibit little or no wear [24], which shows that they avoided biting into bone. In contrast, tyrannosaurids frequently bit in to bone, as demonstrated by spalling of the teeth [26], heavy wear on the tooth apices and the carinae [27], previous identification of bite marks [15] and the presence of bone in a tyrannosaur coprolite [28].

It is usually impossible to refer tooth marks to a particular species, and here, the traces themselves preserve no distinctive features other than their size. However, *Tyrannosaurus* is the only large theropod known from the Late Maastrichtian of the Western Interior [14]. The holotype of “*Nanotyrannus lancensis*” [29] is immature and displays virtually all the features expected for a juvenile *Tyrannosaurus* [14], [30], including a skull with a narrow snout and a broad temporal region, a deep mandible, and an elongate sagittal crest of the frontal [30]. No adults of “*Nanotyrannus*” are known, or juveniles of *T*. *rex* that clearly differ from “*Nanotyrannus*”. Thus, “*Nanotyrannus*” is most parsimoniously considered a juvenile of *Tyrannosaurus*. “*Nanotyrannus*” does have more maxillary teeth than other specimens of *T*. *rex* (fifteen, versus eleven to twelve for other *T*. *rex*) [29], [30], [31] but given that this feature is highly variable within species, and even between the left and right maxillae in a single individual [31], it is insufficient to warrant the recognition of a separate species. Because there is no compelling evidence for more than one tyrannosaurid in the fauna, then by default, the traces described above can be attributed to *Tyrannosaurus*.

Most of the traces described here are smaller than previously described *Tyrannosaurus* tooth marks, which are up to 25 mm in width [15]. This suggests that they were made by juvenile or sub-adult *Tyrannosaurus*, although it is also conceivable that they were made by the smaller posterior teeth of a large individual. However, the broad, shallow tooth marks in MOR 1602 may have been made by a large individual that was not biting at full force.

We argue that these traces result from feeding, rather than intraspecific combat. First, these traces would have been difficult to inflict on a live animal. In the case of MOR 1126, bite marks occur on both the proximal and distal ends of the bone and the shaft, suggesting that the bone was bitten two or three times. It seems unlikely that a small *Tyrannosaurus* would be allowed to repeatedly bite a much larger individual several times on a single toe. In the case of the metatarsal, MOR 1602, the tooth mark runs across the bone's articulation with metatarsal II. Because the metatarsus was tightly bound in life, it would have been difficult to inflict such a mark on the articulated foot of a living animal. Furthermore, fighting animals would be expected to inflict wounds to the head [32] or vulnerable areas such as the neck and flanks, and not the feet or arms. Finally, the absence of healing in any of these specimens is also consistent with the hypothesis that the tooth marks were made on carcasses.

*Tyrannosaurus* therefore seems to have been an indiscriminate and opportunistic feeder, feeding not only on herbivorous dinosaurs, but also on members of its own species. The traces described here likely result from opportunistic scavenging, and were probably made after most of the flesh and organs had been removed from the carcass. Presumably, an animal feeding on a fresh kill would instead be expected to focus on viscera and large muscle masses, which would provide more food with less effort. For feet, toes, and arms to be an appealing source of food, most of the carcass must already have been defleshed. It is somewhat perplexing why so few tooth marks are found on other elements, however. Tooth marks made by Komodo dragons [18] and extant carnivorans [33] tend to be concentrated on elements bearing more meat, and it is therefore surprising not to find more traces made during the initial defleshing of the carcass.

While we interpret these traces as the results of scavenging, we cannot entirely rule out the possibility that these traces result from an individual slowly consuming a kill over an extended period of time. It does seem improbable that *Tyrannosaurus* routinely hunted full-grown members of its own species; however, it is possible that intraspecific combat led to casualties, with the dead becoming a convenient source of food for the victors. Still, compelling evidence for predation in *Tyrannosaurus* remains elusive. Healed injuries in herbivorous dinosaurs are consistent with failed predation [11], [13] but it is debatable whether these traces are actually the results of bites, or some other form of trauma.

Four examples of cannibalism are known from a relatively limited sample of tooth-marked bones. Given this, cannibalism must have been common in *Tyrannosaurus*. If anything, the frequency of cannibalism is easily underestimated, for several reasons. First, the act of feeding on a carcass tends to destroy the evidence, because bones may be ingested, broken up, or dragged off and left to weather away out in the open. Second, cannibalism can only be observed on a carcass where the animals leave tooth marks; where *Tyrannosaurus* fed around the bones, such events would not be recognized. Third, many *Tyrannosaurus* skeletons are mounted, preventing detailed examination of the bones for tooth marks. Fourth, although we examined as many bones in as many museums as possible, it was not possible to examine all specimens of *Tyrannosaurus* in all museums. Given this, it is perhaps surprising to find even a single instance of cannibalism, let alone multiple examples.

Recent studies have questioned whether cannibalism was widespread in dinosaurs [34], but the traces described here show that *Tyrannosaurus* was indisputably a cannibal. The only other dinosaur known to have engaged in cannibalism is the abelisaurid *Majungatholus* [16], however theropod tooth marks also occur on tyrannosaurid bones from the Dinosaur Park Formation [32], [35]. Because two tyrannosaurids- *Gorgosaurus* and *Daspletosaurus*- occur here, it is impossible to definitively state that these traces represent cannibalism [34]. However, because *Gorgosaurus* outnumbers *Daspletosaurus* by three-to-one in this environment [36], most of the bones and feeding traces probably represent *Gorgosaurus* and therefore it is probable that at least some of these traces represent cannibalism.

Cannibalism is common in nature [37], particularly among large carnivores, including bears [38], [39], [40], hyenas [41], large felids [42], [43], Komodo dragons [44], and crocodylians [45], [46]. Notably, most documented cases of cannibalism in large carnivores involve predation, rather than scavenging. Cannibalism is especially common in the American alligator, and may account for more than half of the juvenile mortality each year [46]. Given that cannibalism is known in *Tyrannosaurus*, *Majungatholus* and many extant, large-bodied carnivores, this behavior is likely to have been widespread in large, carnivorous dinosaurs.
